# KCNQ1OT1 aggravates cell proliferation and migration in bladder cancer through modulating miR-145-5p/PCBP2 axis

**DOI:** 10.1186/s12935-019-1039-z

**Published:** 2019-12-03

**Authors:** Jingyu Wang, Hao Zhang, Jie Situ, Mingzhao Li, Hua Sun

**Affiliations:** 1 Department of Urology, Affiliated Hospital of Beihua University, No.12, Jiefangzhong Road, Jilin, 132001 Jilin China; 2 Department of Urology, The Third Affiliated Hospital of Sun Yat-Sen University, Guangzhou, 510000 Guangdong China; 3 Department of Endocrinology, Affiliated Hospital of Beihua University, No.12, Jiefangzhong Road, Jilin, 132001 Jilin China

**Keywords:** KCNQ1OT1, Bladder cancer, Tumorigenesis, miR-145-5p, PCBP2

## Abstract

**Background:**

The large involvement of long non-coding RNAs (LncRNAs) in the biological progression of numerous cancers has been reported. The function of lncRNA KCNQ1OT1 in bladder cancer (BC) remains largely unknown. This study aimed to explore the critical role of KCNQ1OT1 in BC.

**Materials and methods:**

The qRT-PCR was applied to test the expression of RNAs. Cell proliferation was detected by CCK-8 and colony formation assays. Cell apoptosis was measured by TUNEL and flow cytometry experiments. Wound healing and transwell assays were employed to evaluate cell migration and invasion ability respectively. Western blot assay was used to measure relevant protein expression. Immunofluorescence (IF) staining was used to observe EMT process in BC.

**Results:**

KCNQ1OT1 was significantly overexpressed in BC tissue and cell lines. KCNQ1OT1 depletion repressed cell proliferation, migration and invasion, whereas encouraged cell apoptosis. KCNQ1OT1 was a negatively/positively correlated with miR-145-5p/PCBP2 in respect with expression. Mechanically, KCNQ1OT1 was sponge of miR-145-5p and up-regulated the expression of PCBP2. MiR-145-5p inhibition and PCBP2 up-regulation could countervail the tumor-inhibitor role of KCNQ1OT1 knockdown in BC.

**Conclusion:**

KCNQ1OT1 serves as competing endogenous RNA (ceRNA) to up-regulate PCBP2 via sponging miR-145-5p in BC progression.

## Background

On a global scale, bladder cancer (BC) is considered as one of the most common cancers [1]. BC is also considered as the fourth main reason of cancer-associated deaths in males worldwide [2]. Though various therapeutic methods have been utilized in medical treatment, BC patients in advanced stage still confront with poor prognosis outcome [3]. Multifocality, high rates of relapse and lack of sensitive target in early period diagnosis are the major reasons of the poor prognosis of BC [4]. Therefore, it is paramount to find sensitive therapeutic target of BC.

Long non-coding RNAs (lncRNAs) refer to those genes with exceeding 200 nucleotides (nt) in length but without the capacity to encode proteins [5]. LncRNAs have been reported to be abnormally expressed in various cancers, and exerts an irreplaceable function in the carcinogenesis and progression of malignancies [6–11]. LncRNAs are associated with different pathological cellular processes, such as cell proliferation, apoptosis, invasion as well as migration. For example, lncRNA UCA1 facilitates cell growth and migration, yet refrains cell apoptosis in gastric cancer by up-regulating PDL1 through sponging miR-26a/b, miR-193a and miR-214 [12]. LncRNA SNHG1 regulates colorectal cancer epithelial-mesenchymal transition (EMT) process and impacts cell activity via binding with miR-497/miR-195-5p [13]. Hence, targeting lncRNA could hopefully be a theoretical treatment method in cancers.

The prevalent ceRNA hypothesis reveals that lncRNA could influence the function of target gene via competitively binding to miRNA [14, 15]. KCNQ1OT1 is a novel identified lncRNA. Its role in some diseases including cancers has been explored [16, 17]. For example, in colorectal cancer, KCNQ1OT1 serves as ceRNA to facilitate cell migration and EMT process via regulating the expression of miR-217/ZEB1 [18]. While its function in BC still unclear. This study aims to explore whether KCNQ1OT1 functions as ceRNA mechanically in BC tumorigenesis and evolvement, so as to provide a possible target for BC prognosis and treatment.

## Materials and methods

### Human tissue samples

From June 2012 to January 2018, 70 bladder cancer specimens and corresponding normal tissues were gathered from Affiliated Hospital of Beihua University. Patients who provided these tissues did not receive any treatment before being operated. Every patient signed informed consent, based on the review of Ethics of committee of Affiliated Hospital of Beihua University.

### Cell culture

Human bladder epithelial immortalized cell (SV-HUC-1) as well as bladder cancer cells (UM-UC-3, T24, HT-1376 and HT-1197) were provided by Chinese Academy of Sciences (Beijing, China). The above cells were cultured at 37 °C in a moist incubator containing 5% CO_2_ in RPMI-1640 (Invitrogen, Waltham, MA, USA) supplied with 10% FBS (Invitrogen), 1% penicillin (Sigma-Aldrich, Milan, Italy) or streptomycin (Sigma-Aldrich).

### Cell transfection

Transfected cells were put into a 6-well plate until cells was 80% confluence. T24 and HT-1197 cells were co-transfected with shRNAs targeting KCNQ10T1 (sh-KCNQ10T1#1/2), PCBP2 (sh-PCBP2#1/2) and shNC. The pcDNA3.1 vector for PCBP2 was used for overexpression studies. The miR-145-5p mimics, NC mimics and miR-145-5p inhibitor were gained from Genechem (Shanghai China). All plasmids were transfected into cells using Lipofectamine 2000 (Invitrogen).

### Quantitative real-time polymerase chain reaction (qRT-PCR)

Total RNA was isolated from BC cells (T24 and HT-1197) or BC patient tissues according to the reference method TRIzol Reagent (Invitrogen). PrimeScript RT reagent Kit (TaKaRa, Tokyo, Japan) was applied to synthesize cDNA, which was used for qPCR analysis. SYBR Green Master Mix (Applied Biosystems, Foster City, CA, USA) was utilized on 7500 Real-time PCR System (Applied Biosystems), with cDNA as template. The gene expression was calculated by the 2^−ΔΔCt^ method. GAPDH or U6 was the internal control [1].

### Wound healing assay

Cells were placed into 6-well plates at first and cultivated in normal cell growth fluid. Sterile pipette was applied for scratching the cell layer to made scratches. After that, the cells were cleaned and cultured in another medium. Eventually, wound closure was collected at different times.

### Western blot

Total proteins were extracted through a Total Protein Extraction Kit (KeyGen Biotech, Nanjing, China). Concentrations of protein were examined under BCA commercial kit (KeyGen). Proteins were separated using SDS-PAGE and then moved onto PVDF membranes (Millipore, Carlsbad, USA). Membranes were sealed in non-fat milk and then incubated with given primary antibodies: anti-MMP2 (1:1000, ab37150, Abcam, Cambridge, USA), anti-MMP7 (1:1000, ab5706, Abcam), anti-Bcl-2 (1:1000, ab32124, Abcam), anti-bax (1:1000, ab32503, Abcam), anti-caspase 3 (1:1000, ab13847, Abcam), anti-cleaved caspase-3 (1:1000, ab2302, Abcam), anti-caspase 8 (1:1000, ab25901, Abcam), anti-cleaved caspase-8 (1:1000, ab25901, Abcam), anti-caspase 9 (1:1000, ab32539, Abcam), anti-cleaved caspase-9 (1:1000, ab2324, Abcam), anti-E-cadherin (1:1000, ab15148, Abcam), anti-N-cadherin (1:1000, ab76057, Abcam) and anti-GAPDH (1:1000, ab8245, Abcam). GAPDH was used as a measurement control for other proteins. Moreover, the membranes were co-cultured with goat anti-mouse IgG H&L (Cy3^®^) preadsorbed (1:2000, ab97035, Abcam) secondary antibodies for 1 h darkness. Chemiluminescence system (Invitrogen) was employed to observe the protein bands.

### Cell-counting kit 8(CCK-8) and colony formation assays

About CCK-8 assay, cultured T24 and HT-1197 cells were collected to 6-well plates with the density of 1 × 10^4^ cells/well, and then which were mixed with CCK8 reagent (Dojindo, Kumamoto, Japan) for 2 h. The absorption at 450 nm was evaluated at the indicated time points by utilizing microplate reader (EL340; Bio-Tek Instruments, Hopkinton, MA, USA). As for colony formation assay, (800–1000) cells were plated in the six-well plates. Change the medium every 3 days. After 14 days of incubation, PBS (Solibao technology, Shanghai, China) was used to wash and methanol (Grass biotechnology, Nanjing, China) was applied to wash and fix it for 10 min, and then stained with crystal violet (Beyotime Biotechnology, Nantong, China) for 5 min. The visible colony numbers then were counted [19].

### Transwell-invasion assay

T24 and HT-1197 cells in apical compartment were cultured with serum-free medium, and the basolateral chamber was filled with 10% FBS medium. Cells were plated on the top compartment of membrane pre-coated with Matrigel for invasion assay. 24 h later, invaded cells were fixed in methanol (Solarbio, Beijing, China) and dyed in crystal violet (Solarbio). The cell numbers were recorded and photographed randomly from 5 chosen fields by an inverted microscope (Olympus, Tokyo, Japan).

### Flow cytometry assay

Cell apoptosis was evaluated by an annexin/PI Kit (KeyGen). T24 and HT-1197 cells were washed by using PBS (Solarbio) and rehanged, then fixed in 70% cooled ethanol (Solarbio). Ultimately, the rate of apoptosis was analyzed by Flow Cytometer (BD Bioscience, Massachusetts, USA).

### Terminal-deoxynucleoitidyl transferase mediated nick end labeling (TUNEL) assay

TUNEL assays were carried out conforming to supplier’s protocols. After rising twice by PBS, cells of T24 or HT-1197 were fixed and permeabilized by paraformaldehyde (Solarbio) and 0.25% Triton-X 100, respectively. In short, the above cells were stained in DAPI (Invitrogen) or Merge (Invitrogen). Then, cells were surveyed and photographed by fluorescence microscopy (Olympus).

### Subcellular fractionation assay

Subcellular isolation of RNAs in T24 and HT-1197 cells was carried out via Cytoplasmic and Nuclear RNA Purification Kit (Norgenbiotek Corporation, Thorold, Canada). Then the relative expression of KCNQ1OT1 was determined by qRT-PCR. Besides, GAPDH/U6 was regarded as cytoplasmic and nuclear control, separately.

### Fluorescence in situ hybridization (FISH) assay

GenePharma was used to design the FISH probes of KCNQ1OT1. T24 and HT-1197 cells were immobilized for 10 min in 4% PFA and washed with PBS (Sigma-Aldrich) for three times. Subsequently, PBS containing with 0.5% Triton X-100 was applied for cell permeabilization. Following washing, pre-hybridization buffer (Sigma-Aldrich) was added into each well to incubate, which lasted for half an hour. The slide was hybridized with KCNQ1OT1 FISH probe, and then was washed three times with 2× Saline Sodium Citrate (SSC; Sigma-Aldrich) at 42 °C. Hoechst 33342 solution (Invitrogen) was used to stain cells. Finally, fluorescent signal was observed by using the microscope [20].

### Luciferase reporter assay

The wild-type and mutant binding sites of miR-145-5p in KCNQ1OT1 sequence or PCBP2 3′UTR were sub-cloned into pmirGLO dual-luciferase vector to construct plasmids, named KCNQ1OT1-Wt/Mut or PCBP2-Wt/Mut. Later, the plasmids were co-transfected with miR-145-5p mimics into T24 or HT-1197 cells, respectively. At last, the activity of luciferase was examined by Dual-luciferase Reporter System (Promega, Massachusetts, USA). Relative firefly luciferase activity was normalized to Renilla luciferase activity.

### RNA pull down assay

The miR-145-5p-Wt, miR-145-5p-Mut and NC were biotin labeled into Bio-miR-145-5p-Wt, Bio-miR-145-5p-Mut or Bio-NC. Then, cell lysates were cultivated after 48 h and co-incubated with the biotinylated probe and M-280 streptavidin magnetic beads (Sigma). And the level of KCNQ10T1 was analyzed using qRT-PCR subsequently.

### RNA immunoprecipitation (RIP) assay

RIP assays were employed for investigating the potential interaction under a Magna RNA-Binding Protein Immunoprecipitation Kit (Millipore). RNA was extracted via the Total RNA Isolation Kit (Invitrogen), and qRT-PCR was carried out. The lysates were co-cultured in magnetic beads covering anti-Ago2 and IgG in RIP buffer. Eventually, the relative expression quantity was measured by qRT-PCR.

### Tumor growth in nude mice

Approximately 1 × 10^7^ cells were injected into the nude mice subcutaneously. Tumor growth was recorded to measure the width or length, and the volume and weight of the tumor was calculated at indicated times. The experiment was permitted by the ethics of committee.

### Immunofluorescence (IF) staining

T24 or HT-1197 cells were place in 6-well plates and incubated on sterilized coverslips. After cells were treated under different conditions separately, then 4% paraformaldehyde was used to fix cells. Afterwards, 0.5% Triton X-100 was used to make cell permeabilization, which lasted for 10 min at room temperature. Subsequently, 5% BSA (Amersco) was employed to block cells in PBST for one hour, which were incubated with primary antibodies against *E*-cadherin and *N*-cadherin at 4 °C for one whole night, then cultured with fluorochrome-labeled anti-rabbit secondary antibody (MultiSciences), which lasted for one hour at room temperature. Additionally, DAPI (1:5000, Beyotime) was applied to stain the coverslips, which were observed by a fluorescence microscopy (Nikon). Adobe Photoshop 6.0 software was utilized to merge the images.

### Statistical analysis

Data were showed as mean ± SD. SPSS (Chicago, IL, USA) and GraphPad Prism 5 software (San Diego, CA) were applied to make statistical analysis. Significance of the variance between two or several groups was assessed by Student’s t test or ANOVA. Gene expression correlation was conducted by Pearson’s correlation analysis. P value less than 0.05 had statistically significance. The above experiments were made at least thrice.

## Results

### KCNQ1OT1 knockdown inhibits cell proliferation, migration, invasion and EMT in BC

**Fig. 1:**
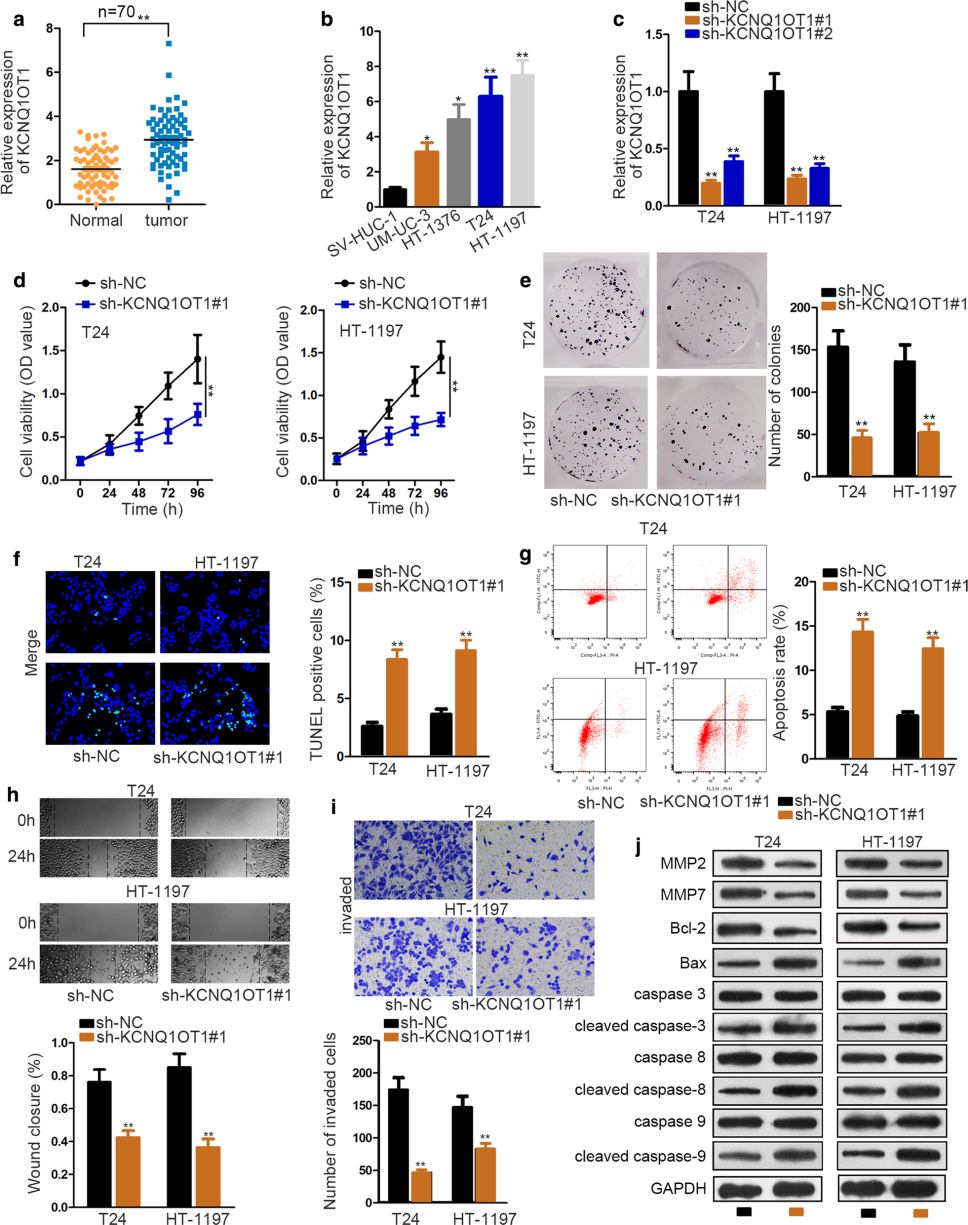
KCNQ1OT1 knockdown inhibits cell proliferation, migration, invasion and EMT in BC. **a**, **b** QRT-PCR evaluated KCNQ1OT1 expression status in BC tissues, cell lines and corresponding matched groups. **c** The transfection efficiency of sh-KCNQ1OT1 plasmid was ensured by qRT-PCR. **d**, **e** CCK-8 and colony formation assays detected sh-KCNQ1OT1-transfected cell proliferation. **f**, **g** TUNEL and flow cytometry assays measured cell apoptosis after knockdown of KCNQ1OT1. **h** Wound healing assay tested sh-KCNQ1OT1-transfected cell migration ability. **i** Transwell assay detected cell invasion after silencing KCNQ1OT1. **j** Western blot measured the level of proteins associated with cell apoptosis and migration after silencing KCNQ1OT1. GAPDH was internal control. ^*^P < 0.05, ^**^P < 0.01

To determine the expression status of KCNQ1OT1 in bladder cancer, 70 BC tissue and adjacent normal tissue specimens were gathered together. Then qRT-PCR result revealed that the expression of KCNQ1OT1 was notably up-regulated in BC tissues (Fig. 1a). Additionally, Further, BC patients with advanced stage (III/IV) possessed more expression than that those with early stage (I/II) (Additional file 1: Figure S1). QRT-PCR also manifested an aberrant overexpression of KCNQ1OT1 in BC cell lines (UM-UC-3, T24, HT‐1376 and HT‐1197) comparing with that in SV-HUC-1 cell (Fig. 1b). Hence, we supposed that it might serve as an oncogene in BC.

To verify the hypothesis, loss-of-function experiments were done in T24 and HT‐1197 cell lines, as these two cell lines contained the most obvious expression of KCNQ1OT1 among the above BC cell lines. Firstly, sh-NC, sh-KCNQ1OT1#1 and sh-KCNQ1OT1#2 plasmids were transfected into T24 and HT-1197 to ensure the transfection efficiency. And qRT-PCR detected that sh-KCNQ1OT1#1 exhibiting a better knockdown effect (Fig. 1c). Therefore, it was chosen for all subsequent assays in need for KCNQ1OT1 inhibition plasmid. CCK-8 and colony formation assays were carried out to detect cell proliferation ability. We observed that knockdown of KCNQ1OT1 significantly crippled BC cell viability (Fig. 1d). The decreased number of colonies illustrated that sh-KCNQ1OT1 inhibited cell proliferation (Fig. 1e). TUNEL examined the influence of sh-KCNQ1OT1 in cell apoptosis. After calculating TUNEL positive rate, we found significantly increased TUNEL positive cells caused by KCNQ1OT1 depletion (Fig. 1f). Also, flow cytometry result validated this conclusion (Fig. 1g). Then wound healing and transwell assays were applied to evaluate the effects brought by sh-KCNQ1OT1 on cell migration and invasion, respectively. We observed that knockdown of KCNQ1OT1 shrank wound closure region and decreased migrated cells dramatically (Fig. 1h, i). Moreover, western blot measured relevant protein expressions. According to result, there was an increased level of Bax and cleaved caspase-3/8/9, while a reduction of MMP2, MMP7 and Bcl-2 after silencing KCNQ1OT1 (Fig. 1j). This further proved that KCNQ1OT1 knockdown promoted cell apoptosis, while curbing cell migration. In addition, KCNQ1OT1 deficiency resulted in rising expression of E-cadherin, and declining expression of N-cadherin, which was illustrated by western blot and immunofluorescence staining assays (Additional file 1: Figure S1B-C). From these findings, we preliminarily judged that KCNQ1OT1 is an oncogene in BC progression.

### KCNQ1OT1 serves as miR-145-5p sponge in BC

**Fig. 2:**
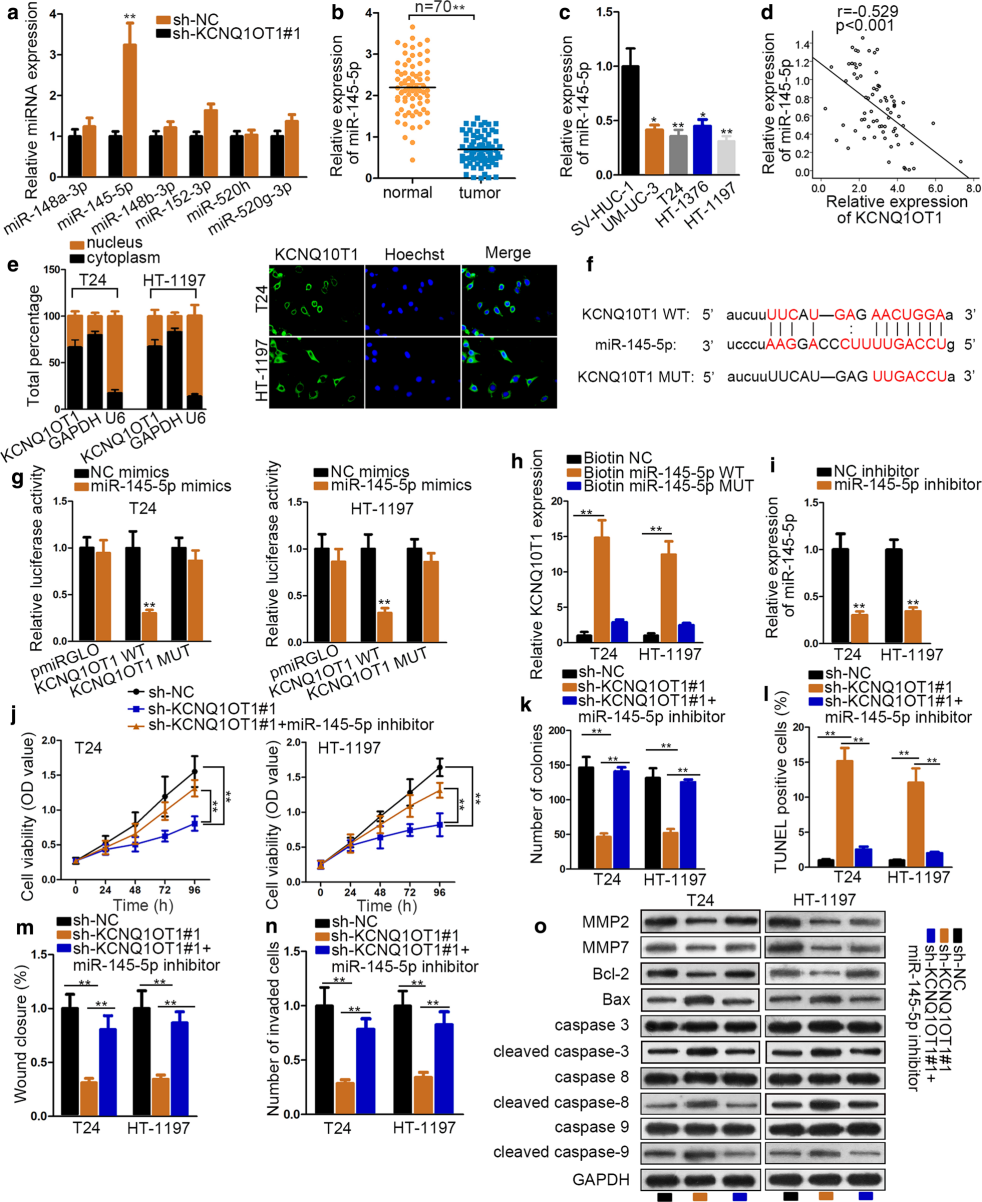
KCNQ1OT1 serves as miR-145-5p sponge in BC. **a** QRT-PCR analysis detected the expression of miRNAs after knockdown of KCNQ1OT1. **b** QRT-PCR assay assessed miR-145-5p expression in BC tissues and normal tissues. **c** QRT-PCR assay examined the expression of miR-145-5p in cell lines. **d** Pearson correlation was conducted to explore the correlation between miR-145-5p and KCNQ1OT1 in BC tissues. **e** Subcellular fractionation and FISH assays determined the distribution of KCNQ1OT1 in nucleus and cytoplasm. GAPDH/U6 was internal control. **f** The binding sites between KCNQ1OT1 and miR-145-5p were predicted by Starbase. **g** Dual luciferase report assay verified the binding relation between KCNQ1OT1 and miR-145-5p. **h** RNA pull down measured the expression of KCNQ1OT1 pulled down by biotin-labeled miR-145-5p group. **i** The transfection efficiency of miR-145-5p inhibitor was guaranteed by qRT-PCR. **j**, **k** CCK-8 and colony formation assays detected cell proliferation in differently transfected groups. **l** TUNEL assay examined cell apoptosis under different transfecting conditions. **m**, **n** Wound healing and transwell assays measured cell migration and invasion respectively among sh-NC, sh-KCNQ1OT1#1 and sh-KCNQ1OT1#1 + miR-145-5p inhibitor groups. **o** Western blot assay measured protein expressions among sh-NC, sh-KCNQ1OT1#1 and sh-KCNQ1OT1#1 + miR-145-5p inhibitor groups. GAPDH was internal control. ^*^P < 0.05, ^**^P < 0.01

Given that lncRNAs usually regulates cancer development via acting as (microRNAs) miRNA sponge [21]. Accordingly, we browsed in Starbase (http://starbase.sysu.edu.cn/) to find combinable miRNAs with KCNQ1OT1. We narrowed scope of the numerous combinable miRNA and found six miRNAs qualified (strict stringency ≥ 5 in Clip data, low stringency in degradome data ≥ 1, and 2 cancer types in Pan-cancer). And qRT-PCR measured that knockdown of KCNQ1OT1 could up-regulate the expression of miR-145-5p most significantly (Fig. 2a), thus we selected miR-145-5p as candidate miRNA for present study. Moreover, significant down-regulated expression of miR-145-5p in BC tissue and cell lines was detected by qRT-PCR (Fig. 2b, c). We also noticed that the expression of miR-145-5p was negatively correlated with that of KCNQ1OT1 from Pearson correlation analysis, which proved the sponge role of KCNQ1OT1 on miR-145-5p (Fig. 2d). Next, subcellular fractionation and FISH assays were employed to determine the location of KCNQ1OT1. Both results manifested that it was principally located in cytoplasm (Fig. 2e). Then, we obtained potential binding sites between miR-145-5p and KCNQ1OT1 from Starbase (Fig. 2f). Dual luciferase reports verified the potential combination between KCNQ1OT1 and miR-145-5p. MiR-145-5p mimics could remarkably attenuate the luciferase activity of KCNQ1OT1 WT compared with negative control groups (Fig. 2g). RNA pull down assay further confirmed this conclusion, as enriched expression of KCNQ1OT1 was pulled down by biotin-miR-145-5p WT group (Fig. 2h). From above findings, we assumed that miR-145-5p could be a tumor suppressor in BC. It was also the upstream gene of KCNQ1OT1. To determine the joint effect on pathological activities, miR-145-5p inhibitor was co-transfected with sh-KCNQ1OT1 in BC cells. Besides, the knockdown efficiency of miR-145-5p inhibitor was ensured by qRT-PCR (Fig. 2i). On one hand, after silencing miR-145-5p, cell viability was significantly increased compared with sh-KCNQ1OT1 group from CCK-8 assay (Fig. 2j). This conclusion was further confirmed by colony formation assay (Fig. 2k). MiR-145-5p inhibitor reversed the pro-apoptosis role of sh-KCNQ1OT1 in BC cells (Fig. 2l). Furthermore, miR-145-5p inhibitor offset the suppressing effects of sh-KCNQ1OT1 on BC cell migration and invasion (Fig. 2m, n). Similarly, western blot assay was measured relevant protein expression. KCNQ1OT1 silencing could increase the level of cell-apoptosis-promoting proteins (Bax and cleaved caspase-3/8/9), but reduced that of cell-apoptosis-suppressing protein (Bcl-2) and cell-migration-promoting proteins (MMP2, MMP7). However, miR-145-5p inhibitor reversed this protein-level-promoting (Bax and cleaved caspase-3/8/9) inhibiting (Bcl-2, MMP2 and MMP7) role of sh-KCNQ1OT1 (Fig. 2o). In a word, KCNQ1OT1 realizes its regulatory effects on BC progression via sponging miR-145-5p.

### MiR-145-5p targets PCBP2 in BC

**Fig. 3:**
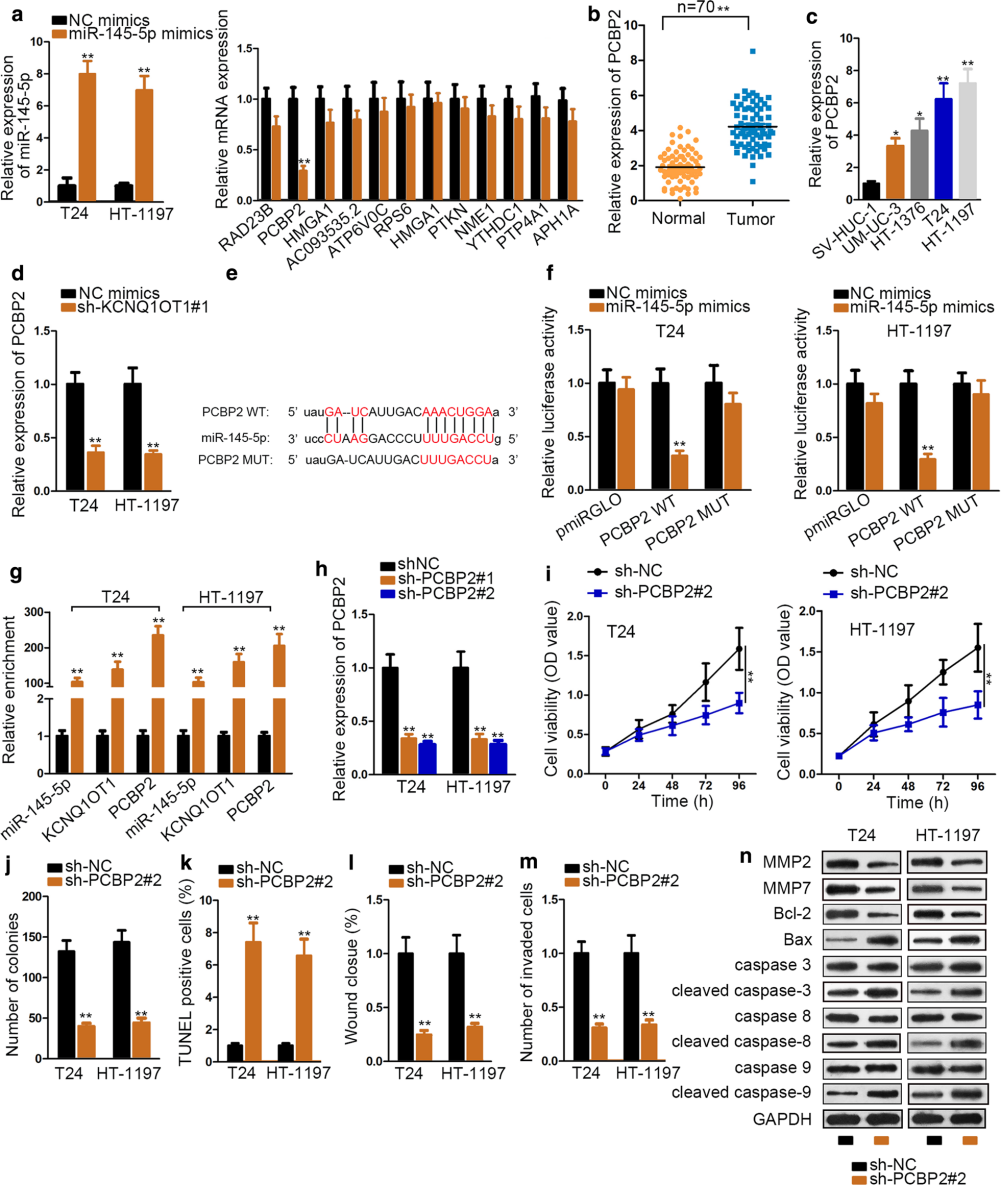
MiR-145-5p targets PCBP2 in BC. **a**, **b** QRT-PCR analysis tested the expression of miR-145-5p and twelve mRNA in miR-145-5p mimics-transfected cells. **b** QRT-PCR assessed PCBP2 expression in BC tissues and matched control groups. **c** QRT-PCR assay detected PCBP2 expression in BC cell lines and SV-HUC-1 cell. **d** QRT-PCR detected PCBP2 expression after knockdown of KCNQ1OT1. **e** There displayed Starbase-predicted binding sites between PCBP2 and miR-145-5p. **f**, **g** Dual luciferase report and RIP assays validated the interactions among KCNQ1OT1, miR-145-5p and PCBP2 was in BC. **h** The transfection efficiency of sh-PCBP2 was guaranteed by qRT-PCR. **i**, **j** CCK-8 and colony formation assays assessed cell proliferation between sh-NC and sh-PCBP2 groups. **k** TUNEL examined cell apoptosis between sh-NC and sh-PCBP2 groups. **l**, **m** Wound healing and transwell assays measured cell migration and invasion between sh-NC and sh-PCBP2 groups. **n** Western blot observed protein level between sh-NC and sh-PCBP2 groups. GAPDH was internal control. ^*^P < 0.05, ^**^P < 0.01

In present study, we proved that KCNQ1OT1 could bind with miR-145-5p, whether this sponge can affect downstream gene of miR-145-5p still needs to be explored. Hence we screened out twelve qualified messenger RNAs (mRNAs) from Starbase (chose strict stringency ≥ 3 on clip data, high stringency on degradome data, 2 cancer types in Pan-cancer and one program for program number). Then we detected which mRNA expression level was influenced most dramatically by miR-145-5p mimics. And the satisfactory overexpression efficiency of miR-145-5p mimics was ensured by qRT-PCR. Subsequently, qRT-PCR measured that over-expression of miR-145-5p caused significant depletion of PCBP2 only, which suggested that PCBP2 could be the most potential target gene (Fig. 3a). To verify this, qRT-PCR detected the relative expression of PCBP2 in BC tissue and cell lines later, and PCBP2 exhibited obviously high expression (Fig. 3b, c). QRT-PCR analysis also showed that knockdown of KCNQ1OT1 can down-regulate the expression of PCBP2 (Fig. 3d). Given this phenomenon, we anticipated that KCNQ1OT1 could regulate the expression of PCBP2 via miR-145-5p. To prove this assumption, we examined whether a chemical interaction existed between miR-145-5p and PCBP2. Hence, the Starbase-predicted binding sites between them were verified by dual luciferase assays result (Fig. 3e, f). We observed miR-145-5p mimics dramatically impaired the luciferase activity of PCBP2 3′-UTR WT compared with negative control groups. After further verification of RIP assay, we obtained the conclusion that PCBP2 physically bound with miR-145-5p (Fig. 3g). Therefore, we proved that KCNQ1OT1 overexpression could upregulate PCBP2 expression via sequestering miR-145-5p in BC.

To elucidate the cellular function of PCBP2, loss-of-function experiments were conducted in T24 and HT-1197 cell lines. The knockdown efficiency of sh-PCBP2 was guaranteed by qRT-PCR assay (Fig. 3h). According to the results of functional experiment, knockdown of PCBP2 repressed cell proliferation, migration and invasion ability, while enhancing cell apoptosis (Fig. 3i–m). Additionally, western blot tested the expression of proteins participating in cell migration and apoptosis, which confirmed the pro-apoptosis and anti-migration role of sh-PCBP2 in BC cells (Fig. 3n). In a summary, PCBP2, the target gene of miR-145-5p, serves as an oncogene in BC progression.

### Overexpression of PCBP2 rescues the tumor-inhibitor role of KCNQ1OT1 depletion in BC

**Fig. 4:**
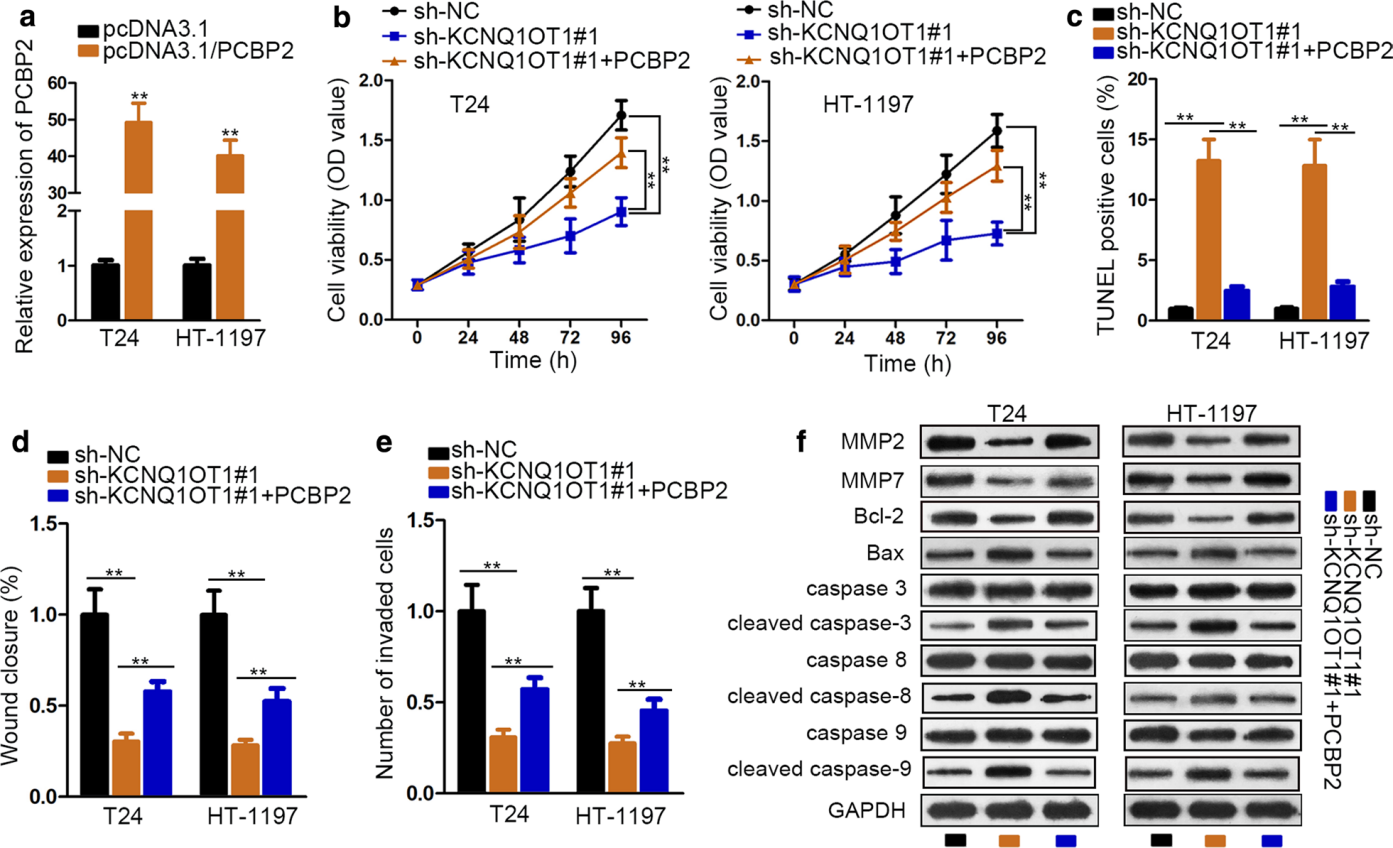
Overexpression of PCBP2 rescues the tumor-inhibitor role of KCNQ1OT1 depletion in BC. **a** The transfection efficiency of pcDNA3.1/PCBP2 was ensured by qRT-PCR. **b** CCK-8 assay evaluated cell viability among sh-NC, sh-KCNQ1OT1#1 and sh-KCNQT1#1 + PCBP2 groups. **c** TUNEL assay examined BC cell apoptosis among sh-NC, sh-KCNQ1OT1#1 and sh-KCNQT1#1 + PCBP2 groups. **d**, **e** Wound healing and transwell assays detected cell migration and invasion respectively among sh-NC, sh-KCNQ1OT1#1 and sh-KCNQT1#1 + PCBP2 groups. **f** Western blot assay evaluated protein level among sh-NC, sh-KCNQ1OT1#1 and sh-KCNQT1#1 + PCBP2 groups. GAPDH was internal control. ^**^P < 0.01

To understand the effect of PCBP2 expression on regulatory mechanism of KCNQ1OT1 in BC, rescue experiments were conducted. The transfection efficiency of pcDNA3.1/PCBP2 was guaranteed by qRT-PCR (Fig. 4a). According to the results of CCK-8, up-regulation of PCBP2 reversed the suppression on cell viability of sh-KCNQ1OT1 (Fig. 4b). In addition, TUNEL assay examined that pcDNA3.1/PCBP2 co-transfected with sh-KCNQ1OT1 could greatly reduce the number of apoptotic cells compared with group treated with sh-KCNQ1OT1 (Fig. 4c). Besides, wound healing and transwell assays measured cell migration and invasion, separately. The wound closure rate of sh-KCNQ1OT1 was obviously lower than negative control group, while pcDNA3.1/PCBP2 remedied this inhibiting effect on cell migration (Fig. 4d). Transwell assay showed that invaded cells decreased remarkably after sh-KCNQ1OT1 transfection compared with negative control group. However, when co-transfecting with pcDNA3.1/PCBP2, the invaded cell number recovered noticeably again (Fig. 4e). The relative proteins expression detected by western blot assay further validate above conclusion (Fig. 4f). In general, PCBP2 upregulation can rescue tumor-inhibitor role of KCNQ1OT1 depletion in BC.

### Knockdown of KCNQ1OT1 hinders xenografts growth in vivo

**Fig. 5:**
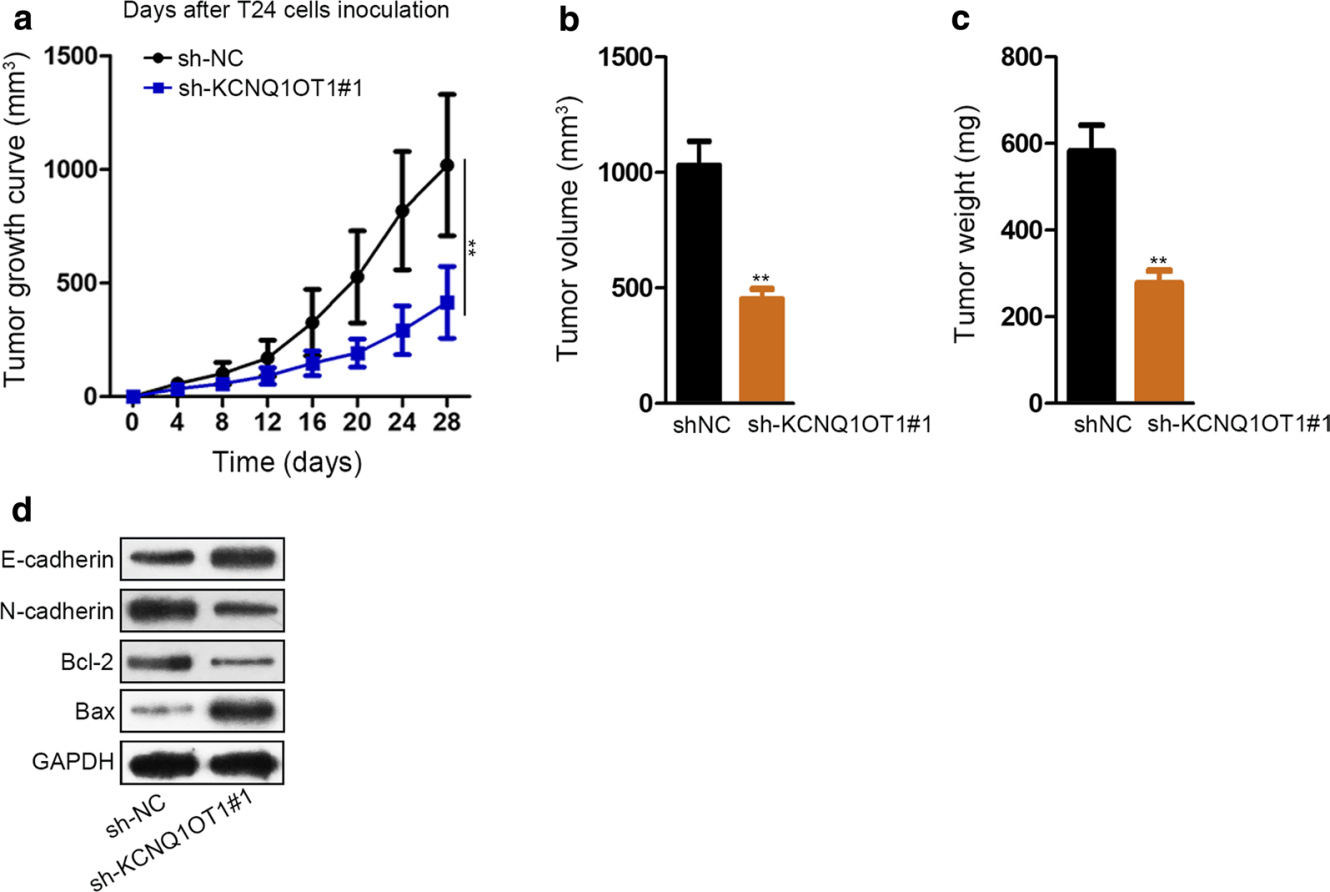
Knockdown of KCNQ1OT1 impedes tumor growth in vivo. **a** The xeograft growth curve between sh-NC and sh-KCNQ1OT1 mice groups. **b** Average tumor volume between sh-NC and sh-KCNQ1OT1 groups was measured after 4-week of indication. **c** Average tumor weight between sh-NC and sh-KCNQ1OT1 was measured at the last day of indicated treatment. **d** Western blot assay measured the expression of *E*-cadherin, *N*-cadherin, Bcl-2 and Bax. GAPDH served as internal control. ^**^P < 0.01

Xenografts with sh-KCNQ1OT1#1 subcutaneously injected on nude mice slowed down the tumor growth compared with negative control (Fig. 5a). The xenograft tumor volume was measured after 4 weeks of injection, and the tumor volume was smaller in sh-KCNQ1OT1 group compared with group injected with sh-negative control (NC) group (Fig. 5b). The tumor weight was also found lighter in sh-KCNQ1OT1 group compared with NC group at the last day of inoculation (Fig. 5c). Western blot also measured that KCNQ1OT1 knockdown inhibited the expression of N-cadherin and Bcl-2, but stimulated that of E-cadherin and Bax in BC tissues (Fig. 5d). These experiments in vivo further proved that KCNQ1OT1 plays an oncogenic role in BC progression.

## Discussion

Long non-coding (lncRNAs) have been widely reported to be related to various human malignancies. Besides, lncRNAs can exert its effects on pathological process through regulating gene transcription and downstream gene expression level [22]. Recently, KCNQ1OT1, as a novel lncRNA, was found to be significantly up-regulated in non-small-cell lung cancer (NSCLC) tissues and cell lines and positively correlated with poor prognosis [23]. Moreover, KCNQ1OT1 was aberrantly highly expressed in breast cancer (BRCA) tissues and cell lines, and it could deteriorate tumor progression via regulating miR-145/CCNE2 expression [24]. Based on these researches, we supposed that KCNQ1OT1 might serve as an oncogene in tumors. In present study, we confirmed that KCNQ1OT1 exhibited abnormally high expression in BC tissues and cell lines, besides, BC patients with advanced stage (III/IV) enjoyed higher level of KCNQ1OT1 than those with early stage (I/II), which was closely associated with tumor cell biological function and pathological processes. Further, knockdown of KCNQ1OT1 dampened cell proliferation, migration, invasion and EMT, while expedited cell apoptosis. Silencing KCNQ1OT1 also repressed xenografts growth in vivo. All these findings indicated that KCNQ1OT1 is an oncogene in BC progression.

To date, accumulating studies have found that lncRNA could act as a competing endogenous RNA (ceRNA) of microRNA (miRNA) and restore the miRNA downstream target gene function to a large extent. For instance, lncRNA WDFY3-AS2 can regulate the expression of RORA via sponging miR-18a in ovarian cancer [25]. Also, in diabetic cardiomyopathy (DCM), lncRNA DCRF can up-regulate PCDH17 expression through sponging miR-551b-5p, leading to increased cardiomyocyte autophagy in DCM [26]. Likewise, some lncRNAs involved in the tumorigenesis of BC have been reported as well. For example, lncRNA OXCT1-AS1 could bind with miR-455-5p and decrease the combination of miR-455-5p with JAK1, thus increasing expression of JAK1 in BC [27]. MiR-145-5p, a target of KCNQ1OT1, was found to be significantly down-regulated and was a tumor suppressor in some tumors [28, 29]. In this study, we also noticed a remarkable down-regulation of miR-145-5p in BC tissues and cell lines. We further proved that KCNQ1OT1 mainly located in cytoplasm was an effective sponge of miR-145-5p. Moreover, the interactions between KCNQ1OT1 and miR-145-5p via luciferase activity report and RNA pull down assays. Additionally, miR-145-5p suppression counteracted the inhibiting influence of KCNQ1OT1 knockdown in BC cell proliferation, migration and invasion.

Further, we selected PCBP2 as candidate target gene of miR-145-5p as it exhibited dramatically the lowest expression among the twelve predicted combinable genes after up-regulation of miR-145-5p. PCBP2 was reported to play a tumor facilitator role in several cancers. For example, PCBP2 expression was up-regulated in pancreatic ductal adenocarcinoma (PDAC) and was correlated with advanced PDAC stages as well as poor diagnosis [30]. In present study, we observed that the expression of PCBP2 was also up-regulated in BC tissues and cell lines. PCBP2 depletion inhibited cell proliferation, migration and invasion, but stimulated cell apoptosis in BC. In addition, overexpression of PCBP2 reversed the tumor-suppressing function caused by sh-KCNQ1OT in BC. More specifically, KCNQ1OT1 overexpression can aggravate cell proliferation, migration and invasion yet inhibit cell apoptosis via up-regulating PCBP2 expression as miR-145-5p sponge in BC.

## Conclusion

Our study suggests the KCNQ1OT1/miR-145-5p/PCBP2 axis existing in BC for the very first time. We identified KCNQ1OT1 a tumor facilitator in BC development and it could hopefully become a diagnosis biomarker and therapeutic strategy for BC patients.

## Supplementary information

**Additional file 1: Figure S1.** A. QRT-PCR measured the expression of KCNQ1OT1 in BC patients at early (I/II) and advanced stages (III/IV). (B-C) Western blot and Immunofluorescence (IF) assays separately measured the expression and fluorescence intensity of E-cadherin and N-cadherin. GAPDH was used as internal control. **P < 0.01.

## Data Availability

Research data and material are not shared.

## Abbreviations

BC: bladder cancer
BRCA: breast cancer
CCK-8: cell-counting kit 8
ceRNA: competing endogenous RNA
DCM: diabetic cardiomyopathy
EMT: epithelial-mesenchymal transition
FISH: fluorescence in situ hybridization
IF: immunofluorescence
lncRNAs: long non-coding RNAs
mRNAs: messenger RNAs
miRNAs: micro RNAs
NC: negative control
NSCLC: non-small-cell lung cancer
PDAC: pancreatic ductal adenocarcinoma
qRT-PCR: quantitative real-time PCR
RIP: rNA immunoprecipitation
TUNEL: terminal-deoxynucleoitidyl transferase mediated nick end labeling
