# Supplementary figures and images for: KCNQ1OT1 aggravates cell proliferation and migration in bladder cancer through modulating miR-145-5p/PCBP2 axis
Source: Cancer Cell Int. 2019 Dec 3;19:325. doi: 10.1186/s12935-019-1039-z (PMC6889643; doi:10.1186/s12935-019-1039-z)

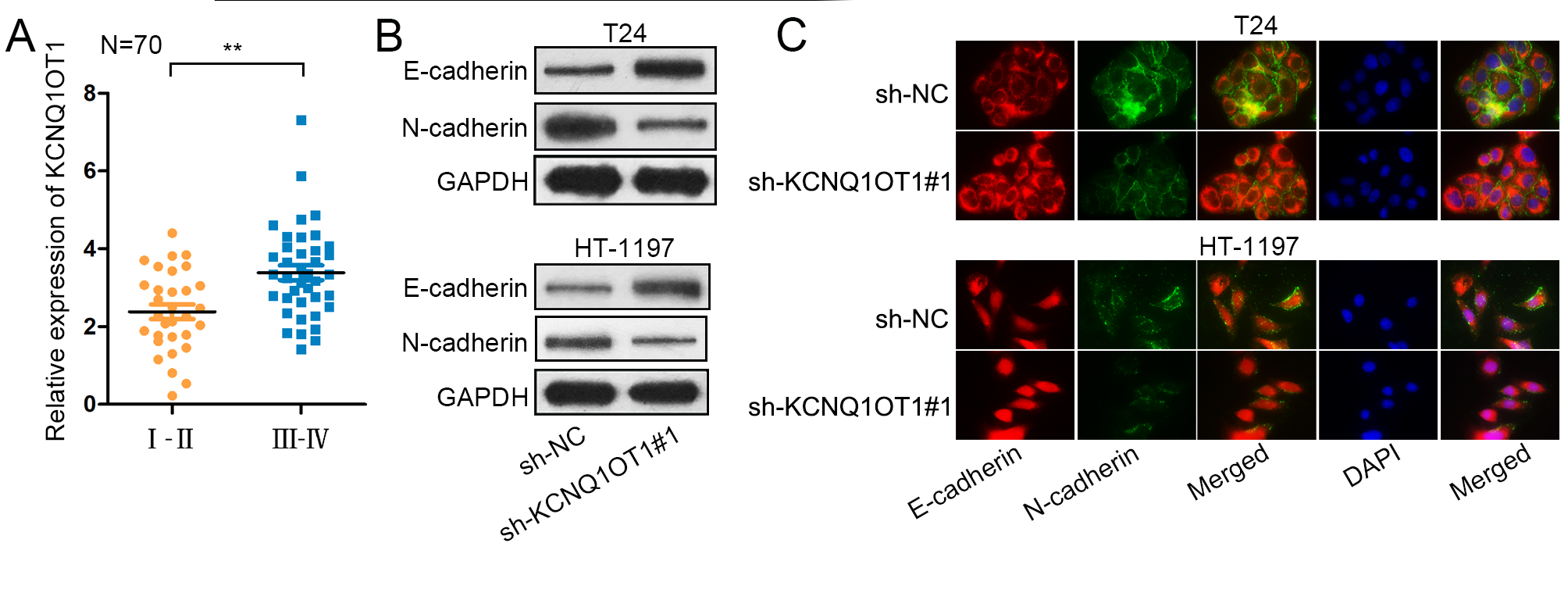

Supplement: Supplementary file 1 — Additional file 1: Figure S1. A. QRT-PCR measured the expression of KCNQ1OT1 in BC patients at early (I/II) and advanced stages (III/IV). (B-C) Western blot and Immunofluorescence (IF) assays separately measured the expression and fluorescence intensity of E-cadherin and N-cadherin. GAPDH was used as internal control. **P < 0.01. [file 12935_2019_1039_MOESM1_ESM.tif]
